# Natural killer cells expanded *in vivo* or *ex vivo* with IL-15 overcomes the inherent susceptibility of CAST mice to lethal infection with orthopoxviruses

**DOI:** 10.1371/journal.ppat.1008505

**Published:** 2020-04-22

**Authors:** Patricia L. Earl, Jeffrey L. Americo, Bernard Moss

**Affiliations:** Laboratory of Viral Diseases, National Institute of Allergy and Infectious Diseases, National Institutes of Health, Bethesda, Maryland, United States of America; Thomas Jefferson University, UNITED STATES

## Abstract

The wild-derived inbred CAST/EiJ mouse, one of eight founder strains in the Collaborative Cross panel, is an exceptional model for studying monkeypox virus (MPXV), an emerging human pathogen, and other orthopoxviruses including vaccinia virus (VACV). Previous studies suggested that the extreme susceptibility of the CAST mouse to orthopoxviruses is due to an insufficient innate immune response. Here, we focused on the low number of natural killer (NK) cells in the naïve CAST mouse as a contributing factor to this condition. Administration of IL-15 to CAST mice transiently increased NK and CD8^+^ T cells that could express IFN-γ, indicating that the progenitor cells were capable of responding to cytokines. However, the number of NK cells rapidly declined indicating a defect in their homeostasis. Furthermore, IL-15-treated mice were protected from an otherwise lethal challenge with VACV or MPXV. IL-15 decreased virus spread and delayed death even when CD4^+^/CD8^+^ T cells were depleted with antibody, supporting an early protective role of the expanded NK cells. Purified splenic NK cells from CAST mice proliferated *in vitro* in response to IL-15 and could be activated with IL-12/IL-18 to secrete interferon-γ. Passive transfer of non-activated or activated CAST NK cells reduced VACV spread but only the latter completely prevented death at the virus dose used. Moreover, antibodies to interferon-γ abrogated the protection by activated NK cells. Thus, the inherent susceptibility of CAST mice to orthopoxviruses can be explained by a low level of NK cells and this vulnerability can be overcome either by expanding their NK cells *in vivo* with IL-15 or by passive transfer of purified NK cells that were expanded and activated *in vitro*.

## Author summary

With the eradication of smallpox, monkeypox virus (MPXV) remains the only poxvirus causing significant mortality in humans. Although endemic in parts of Africa, human infections have occurred in the United States, the United Kingdom and Israel due to travelers or imported animals. Contrary to its name, MPXV primarily infects rodents and secondarily infects humans and other primates. The wild-derived CAST mouse is an excellent small animal model for studying the pathogenicity of MPXV and related orthopoxviruses including vaccinia virus (VACV) and for evaluating therapeutics. We previously found that the susceptibility of CAST mice is correlated with low numbers of natural killer (NK) cells and a delayed interferon-γ response. Here we showed that *in vivo* administration of the cytokine IL-15 transiently raised NK cell numbers and protected CAST mice from systemic infections with VACV and MPXV. CAST mouse NK cells that were purified and expanded *in vitro* with IL-15 also provided protection, further demonstrating the important role of NK cells. The rapid decline in NK cell numbers following cessation of IL-15 administration or NK cell transfer suggests that a low level of NK cell homeostasis contributes to the susceptibility of CAST mice to virus infection.

## Introduction

The orthopoxviruses (OPXVs) comprise a large and well-studied genus of poxviruses, two members of which cause lethal human disease: variola virus (VARV) and monkeypox virus (MPXV), the causative agents of smallpox and an emerging smallpox-like disease, respectively [1]. The potential use of either virus for terrorism has led to their classification by the United States as Select Agents (https://www.selectagents.gov). Although VARV has been eradicated from nature, MPXV is endemic in Africa with an increasing incidence of human infections [2–5]. In addition, MPXV was imported into the United States via rodents [6, 7] and into the United Kingdom [8] and Israel (https://www.health.gov.il/English/News_and_Events/Spokespersons_Messages/Pages/12102018_1.aspx) by international travelers, raising the specter of an expanding geographic range. Human monkeypox differs from smallpox in having a lower but still significant mortality rate cited as high as 11% [9] and substantial but less frequent human-to-human transmission [10]. There are at least two strains of MPXV from different geographic areas that produce different disease outcomes in humans, although the genetic basis for the latter has not been established [11, 12]. Unlike VARV, MPXV is disseminated in rodent reservoirs and therefore unlikely to be eradicated.

Several animal models are under investigation to learn more about MPXV virulence. Despite its name, which reflects the discovery of MPXV in captive monkeys, the latter are not a major reservoir for MPXV and large doses are required to achieve clinical disease [13]. As rodents appear to be natural hosts of MPXV, a variety of such animals including ground squirrels [14, 15], black tailed prairie dogs [16–19] and African dormice [20] have been investigated as models. Nevertheless, these experimental systems have drawbacks because of the unavailability of commercial breeding, genetic heterogeneity and absence of immunological and other reagents. The common laboratory inbred strains of mice, for which immunological reagents are available, are resistant to MPXV disease unless severely diminished in innate or acquired immunity by genetic or chemical means [21–23]. The extreme susceptibility of an inbred strain of Mus musculus castaneus (CAST) to MPXV is exceptional and was discovered during a screen of 38 mouse strains, which revealed that only three acquired a lethal infection [24]. The three susceptible mouse strains are each wild-derived and of these CAST/EiJ is most sensitive to MPXV with lethal dose 50 (LD_50_) values of 680 and 14 infectious units via intranasal (IN) and intraperitoneal (IP) routes of infection, respectively, whereas no deaths occur in the common inbred strains at the highest tested dose (10^7^ infectious units). A less virulent West African-derived MPXV strain exhibits a LD_50_ one log higher in the CAST mouse, suggesting that the latter will be useful in investigating virulence. Indeed, the CAST mouse was cited in a comprehensive review as the best small animal model for MPXV infection [25]. Subsequent studies showed that CASA, an independently derived inbred strain of Mus musculus castaneus, was similarly susceptible to MPXV [26].

CAST and CASA mice were derived from Southeastern Asian house mice trapped in Thailand. CAST, because of extensive genetic differences from other inbred mice, was chosen as one of the eight founder strains of the Collaborative Cross panel [27–29]. In addition to MPXV, CAST mice are exceptionally susceptible to vaccinia virus (VACV) and cowpox virus (CPXV) [30] as well as to influenza virus [31], indicating that they may provide a model for research on multiple pathogens and provide new knowledge of virus-host interactions. For both OPXV and influenza virus infections, the vulnerability of CAST mice is associated with high viral loads and low numbers of NK cells. Although highly susceptible to primary OPXV infection, CAST mice mount effective humoral and T cell responses when immunized with an attenuated vaccine strain of VACV and are fully protected against a subsequent MPXV challenge [24]. In accord with the idea that the sensitivity of the CAST mouse is due to an insufficient innate immune response, increases in interferon-γ (IFN-γ) and tumor necrosis factor-α are delayed and lower in CAST mice compared to BALB/c mice following OPXV infection or poly(I:C) inoculation [32, 33]. Additionally, naïve CAST mice are protected by exogenously administered IFN-γ, which provides an extended survival period for adaptive immunity to develop and eventually clear the virus. Since NK cells are major early producers of IFN-γ, we conjectured that the susceptibility of CAST mice to OPXV infection may be due to low NK cell numbers or a defect in their function. In this regard, some previous studies showed that depleting NK cells of C57BL/6 mice enhances OPXV virulence [34]. To better understand the nature of the NK cell deficiency in CAST mice, we posed the following questions: (i) can the NK cells of CAST mice be expanded by administration *in vivo* of IL-15, a gamma chain cytokine that signals through a trimeric receptor and is a known mediator of NK cell homeostasis [35–39]; (ii) would IL-15-treated mice be protected against lethal challenges with OPXV; (iii) can CAST mouse NK cells be expanded *in vitro* with IL-15 and activated by IL-12 and IL-18 to produce IFN-γ and (iv) would activated NK cells provide enhanced protection upon passive transfer? Answers to these and additional questions are provided below. Because the susceptibility of CAST mice to MPXV and VACV are similar and MPXV is a Select Agent requiring a high level of containment, the majority of studies here were carried out with VACV.

## Results

### IL-15 increases NK and CD8^+^ T cell numbers in CAST mice

**Fig 1:**
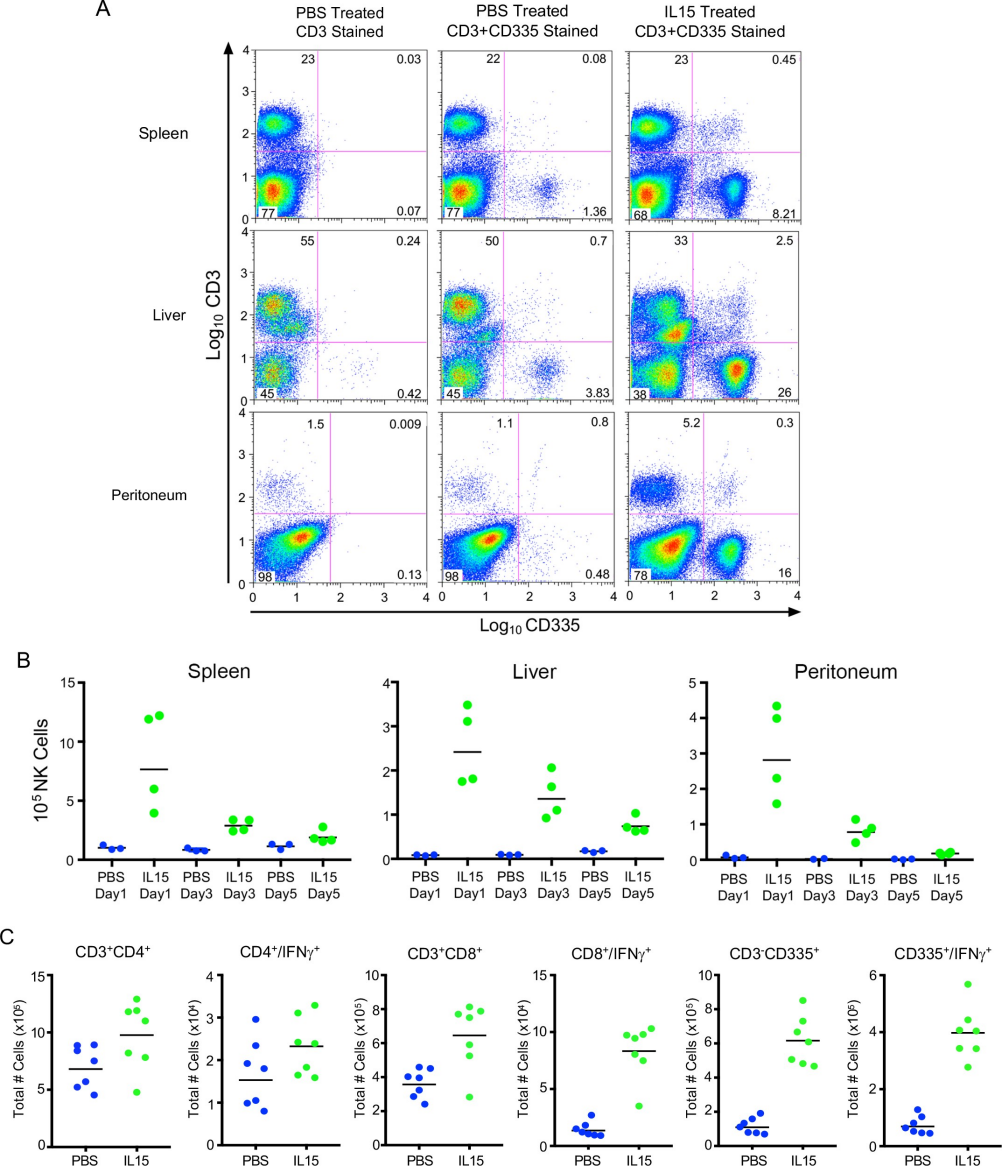
Proliferation of NK cells following IL-15 treatment of CAST mice. **(A)** NK cell numbers determined by flow cytometry. Mice were inoculated IP with PBS or PBS containing 5 μg of IL-15 daily for four days. On the following day, cells from spleens, livers and peritoneal washes were stained with fluorescein isothiocyanate (FITC)-conjugated anti-CD3 and allophycocyanin (APC)-conjugated anti-CD335 antibodies and analyzed by flow cytometry. To identify NK cells, PBS-treated samples were stained with anti-CD3 alone and gating tools were used to partition CD335^+^ cells in the upper-right quadrant of the scatter plot (first column). The established gates were then applied to all IL-15- and PBS-treated samples stained with both anti-CD3 and anti-CD335. **(B)** Decrease in NK cell numbers with time. NK cells were analyzed as in panel A at 1, 3 and 5 days after cessation of IL-15 inoculations and the numbers for individual mice are shown. Bars indicate geometric mean. Comparison of NK cell numbers in spleen and liver of PBS- and IL-15-treated mice on days 1, 3 and 5 and in peritoneal washes on days 1 and 5 were significant (p = 0.057). **(C)** Enumeration of IFN-γ expressing NK and T cells. Mice were inoculated IP with PBS or PBS containing 5 μg of IL-15 daily for four days and splenic cells were isolated and stimulated with PMA/Ionomycin for 4 h, treated with Brefeldin A and stained intracellularly with mouse anti-IFN-γ -APC antibody. Surface staining was accomplished with anti-CD3-FITC, anti-CD4-PE, anti-CD8-PerCP and anti-CD335-PerCP. Increases in NK cells following IL-15 were shown in three independent experiments.

The observation that NK cell numbers are lower in CAST mice compared to classical inbred strains suggests either a deficiency in their cellular development, proliferation or maintenance in the periphery, which could result from inadequate growth factors, receptors or signaling pathways. IL-15 has been shown to increase and sustain NK and CD8^+^ T cell numbers in C57BL/6 mice [40, 41]. To determine whether production of CAST NK and T cells would be stimulated by IL-15, we administered phosphate buffered saline (PBS) without or with 5 μg of recombinant murine IL-15 by the IP route on four successive days, after which lymphocytes were harvested from the spleen, liver and peritoneal cavity and analyzed by flow cytometry. NK cells were identified as CD3^-^ and bearing the CD335^+^ (NKp46) antigen as shown in the scatter plots for the various organs on day 1 post-IL-15 (Fig 1A). Quantitation of total NK cells on days 1, 3 and 5 following IP inoculation of PBS or IL-15 are shown in Fig 1B. Following IL-15 treatment, NK cell numbers were dramatically higher on day 1 but progressively decreased over the next 4 days, although remaining significant (p = 0.057) relative to the PBS control throughout (Fig 1B).

Previous studies had shown extremely low numbers of NKT cells in CAST mice compared to classical inbred strains [42]. Indeed, of the eight Collaborative Cross strains, CAST mice had the lowest number of NKT cells (0.060% and 0.067% of total spleen lymphocytes for males and females, respectively). We found similarly low numbers of NKT (CD3^+^CD1d^+^) cells in the spleen, which were not increased by IL-15 (S1 Fig). The percentage of NKT cells in IL-15-treated mice actually decreased because of increases of other CD3^+^ lymphocytes. The majority (65%) of NK cells from IL-15-treated CAST mice were capable of expressing IFN-γ, whereas only 13% of CD8^+^ T cells and 2.5% of CD4^+^ T cells could express IFN-γ (Fig 1C).

In summary, administration of IL-15 greatly increased the number of NK cells and to a lesser extent CD4^+^ and CD8^+^ T cells suggesting that the low numbers in naïve mice are not due to an inability of IL-15 to interact with receptors on progenitor cells and induce proliferation. Furthermore, the NK cells could express IFN-γ indicating that they were activated by endogenous cytokines. However, the NK cell numbers rapidly decreased following cessation of IL-15 administration suggesting low NK cell homeostasis in the periphery.

### IL-15 overcomes the susceptibility of CAST mice to lethal infections with VACV and MPXV

**Fig 2:**
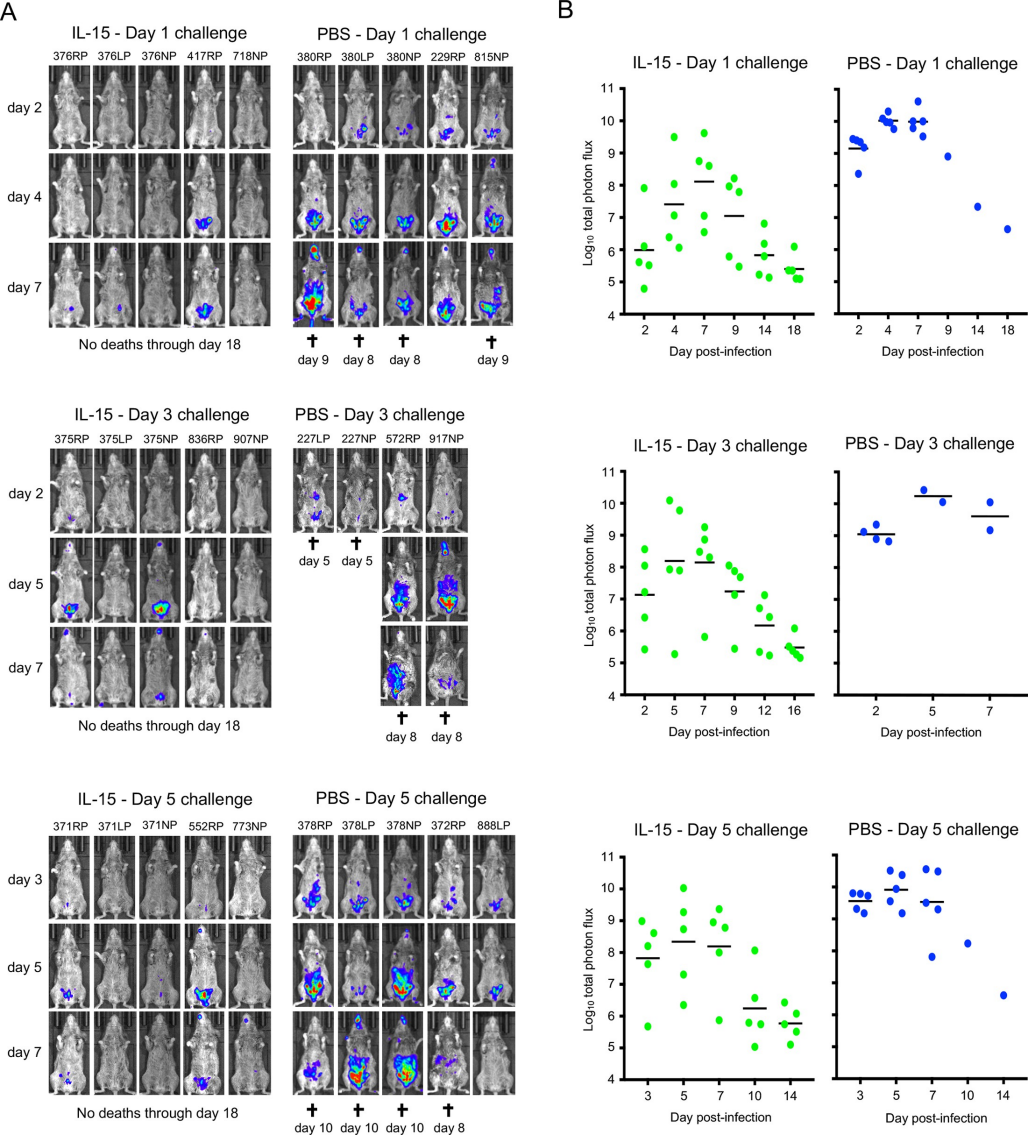
VACV challenge of IL-15-treated CAST mice. **(A)** CAST mice were treated with IL-15 or PBS as in Fig 1 and challenged IP with 460 PFU of VACV expressing FLuc on days 1, 3 and 5 after cessation of IL-15 administration. The progress of infection was measured by injecting luciferin and measuring luminescence. Ventral view images of surviving mice are shown on the indicated day post-infection using the same exposure time and binning factor, which was insufficient to show the low levels of luminescence seen at higher exposure in some mice. Red, blue and purple denote intensity of luminescence from high to low. Days of death or euthanasia (†) are indicated. **(B)** Total photon flux (photons per second per square centimeter per steradian) of the entire mouse was calculated and shown for each individual. Bars indicate geometric means. Protection of IL-15 treated mice was shown in three independent experiments.

Since IL-15 treatment increased the numbers of NK and CD8^+^ T cells, we investigated whether the treated CAST mice would be protected against an OPXV challenge. OPXV challenges are frequently administered by the IN or IP route. The IP route was chosen here based on previous studies with CAST mice that correlated spread from the respiratory tract to the abdominal region with lethality and a lower LD_50_ for IP compared to IN [33]. Measurement of virus spread in live mice was facilitated by using recombinant viruses that express firefly luciferase (FLuc), which does not significantly attenuate either VACV or MPXV [30, 43]. In the following experiment, mice were challenged with VACV strain WR expressing FLuc (WRvFire) at 1, 3 or 5 days after administration of PBS or PBS with IL-15 and then imaged over the next 18 days. Because of the large number of animals involved, the treatments and challenges were staggered but always included PBS as well as IL-15 groups at each time point. All IL-15-treated mice survived for the duration of the experiment regardless of whether they were challenged 1, 3 or 5 days after cessation of IL-15 treatment, whereas all but 2 of 14 control mice succumbed 8–10 days after challenge. In the controls, luminescence was most intense in the abdominal area but was also detected in the heads of some mice after several days (Fig 2A) and the photon flux peaked between days 4 to 8 (Fig 2B). Luminescence in the IL-15-treated mice also increased during this time but was much lower in intensity than in the control groups (p<0.01 after day 1 and 5 challenges; p = 0.056 after day 3 challenge calculated prior to any animal deaths) and diminished subsequently (Fig 2B). Note that for comparative purposes, all images were prepared using the same luminescence exposure time and binning factor, which was insufficient to show the low levels of luminescence that could be seen at higher exposure in some mice. However, even low levels of luminescence are quantitated in the total photon flux. In this and other experiments, we sometimes noted a decrease in luminescence prior to death of the animals. Luminescence results from a dynamic process dependent on active synthesis of luciferase and uptake of luciferin, which may decline following severe infection and cellular necrosis.

The ability of IL-15 treatment to protect CAST mice against challenge by a FLuc expressing derivative of the pathogenic MPXV Zaire-1979 strain (MPXV-Z06) was also demonstrated. For this experiment, CAST mice were treated with PBS alone or with IL-15 for 4 days and challenged with MPXV on the following day. As with VACV, the IL-15-treated mice had lower luminescence and total photon flux compared to the controls (p = 0.0286 on days 4 and 7) and all survived whereas 3 of 4 control mice died between days 7 to 9 (S2A and S2B Fig).

### Resistance of IL-15-treated CAST mice during first week of VACV infection is independent of CD4^+^/CD8^+^ T cells

**Fig 3:**
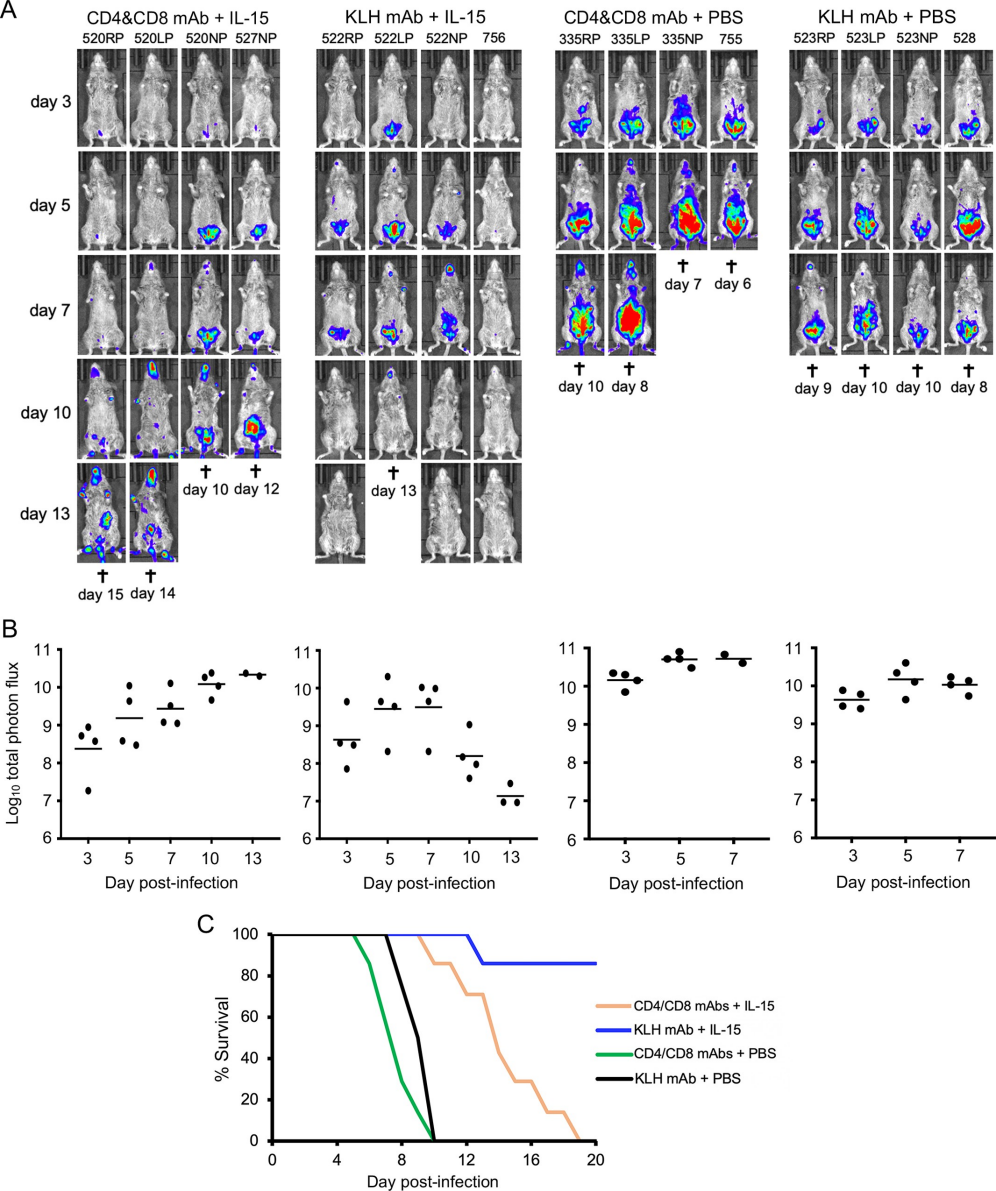
Resistance of IL-15-treated CAST mice during first week of VACV infection is independent of CD4^+^/CD8^+^ T-cells. **(A)** CAST mice were treated with PBS or IL-15 and received either anti-CD4 and anti-CD8 antibodies to deplete T-cells or anti-KLH mAb as a control. The mice were infected with 590 PFU of VACV expressing FLuc and the progress of infection was monitored by measuring luminescence on the indicated days as described in the legend to Fig 2A. **(B)** Plots of total photon flux of individual animals are below the images for each group. **(C)** Days of death or euthanasia (†) are indicated and plotted as survival curves. IL-15-protection of mice treated with anti-CD4 and CD8 mAbs was shown in two independent experiments. Bars indicate geometric means.

Complete protection of mice against an OPXV challenge depends on both innate and adaptive immune responses [44]. Since NK and CD8^+^ T cells were elevated following IL-15 treatment of CAST mice, it was important to determine whether both are necessary for early control of the infection. Preliminary experiments confirmed >95% depletion of CD4^+^ and CD8^+^ cells in CAST mice treated with anti-CD4/CD8 mAbs. CAST mice were divided into four groups each of which received IL-15 or PBS and anti-CD4/CD8 mAbs or anti-KLH isotype control mAb. Luminescence was not significantly different (p = 0.9) for the first 7 days in mice that received IL-15 and were depleted or not depleted of CD4^+^/CD8^+^ T cells (Fig 3A and 3B). This result indicated that T cells were not required for the protective effect during the first week after challenge. However, T cells were required for virus clearance during the second week of infection as luminescence in the depleted mice continued to rise, whereas luminescence diminished in the mice that received anti-KLH mAb and were not T cell depleted. Although the combined mice (n = 7) of this experiment and a repeat that had been treated with IL-15 and T cell-depleted succumbed to the infection, the time to death was prolonged (median 14 days) compared to a median of 8 days for T-cell depleted mice that did not receive IL-15 (p<0.0001, Mantel Cox) (Fig 3C). Taken together these data indicate that the IL-15-induced NK cells were important for limiting virus spread before development of adaptive immunity in CAST mice.

### IL-15 receptor-α knock-out mice exhibit increased susceptibility to VACV

**Fig 4:**
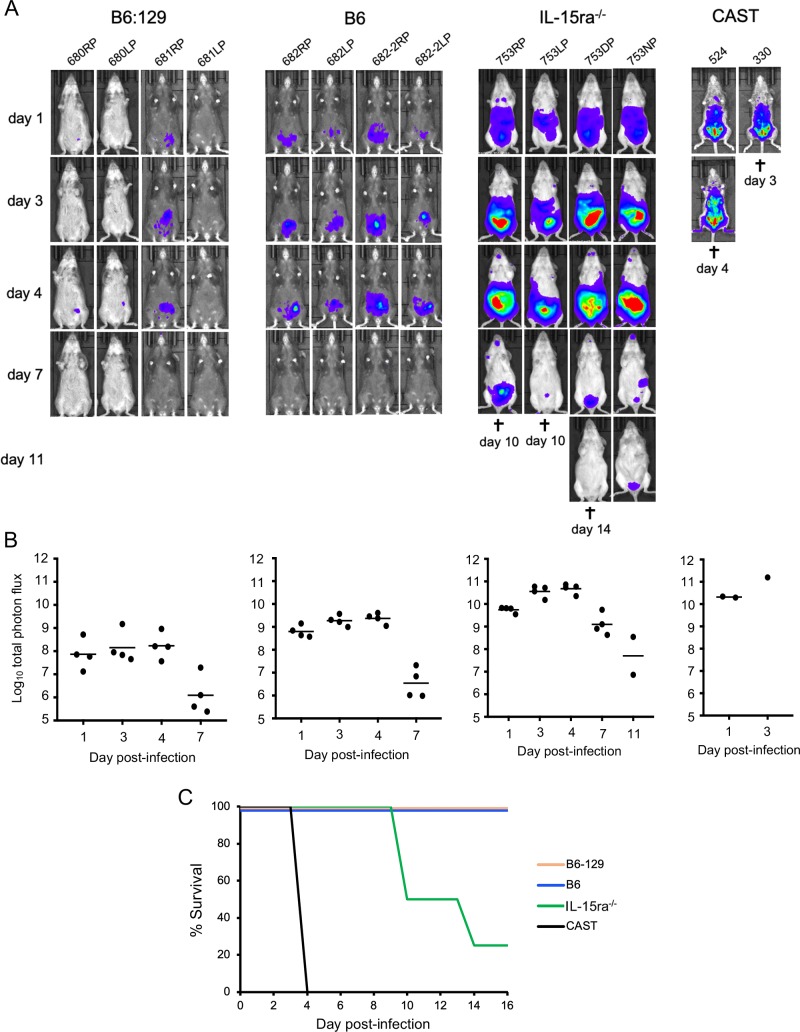
IL-15 receptor α knock-out mice exhibit increased susceptibility to VACV. **(A)** IL-15ra^-/-^ (B6:129X1-*Il15ra*^*tm1Ama*^/J), B6 (C57BL/6J), B6:129 (F1 hybrid of B6 female and 129 male) and CAST mice were inoculated IP with 10^6^ PFU of VACV expressing FLuc. Luminescence was measured as described in the legend to Fig 2A and ventral images taken on successive days. Note that half of the hybrid mice are black. **(B)** The plots of total photon flux of individual animals are below the images for each group. **(C)** Days of death (†) or euthanasia are indicated and plotted as survival curves. Bars indicate geometric means.

We were interested in comparing the susceptibility of CAST mice to VACV infection with another mouse strain deficient in NK cells. For this purpose, IL-15ra^-/-^ mice (B6:129X1-*Il15ra*^*tm1Ama*^/J), which have deficiencies of NK and CD8^+^ T cells [45] were used. CAST mice, IL-15ra^-/-^ mice, and control parental strains C57BL/6J and B6:129 X1 were inoculated IP with 10^6^ PFU of VACV. The high virus dose was used because of the inherent resistance of classical inbred strains to VACV [24]. Luminescence intensity and total photon flux were higher (p = 0.0286) in the IL-15ra^-/-^ mice than the two control parental strains on days 1–7 (Fig 4A and 4B), similar to the difference that we saw between untreated and IL-15-treated CAST mice albeit infected with a lower dose of VACV (Fig 2A). Although luminescence of the IL-15ra^-/-^ mice started to decline on day 7, three of the four succumbed to the infection between days 10 and 14 (Fig 4C). At this high 10^6^ PFU dose of VACV, CAST mice expired within 4 days as previously reported [33] and confirmed here (Fig 4C). The time to death of the IL-15ra^-/-^ mice was longer than for the CAST mice, which is attributable to the intrinsic resistance of the parental strains to VACV. In this context, we previously provided evidence that the greater resistance of C57BL/6J mice to infection with OPXV compared to CAST mice is due to multiple gene loci [26].

### Purification and expansion of splenic NK cells

**Fig 5:**
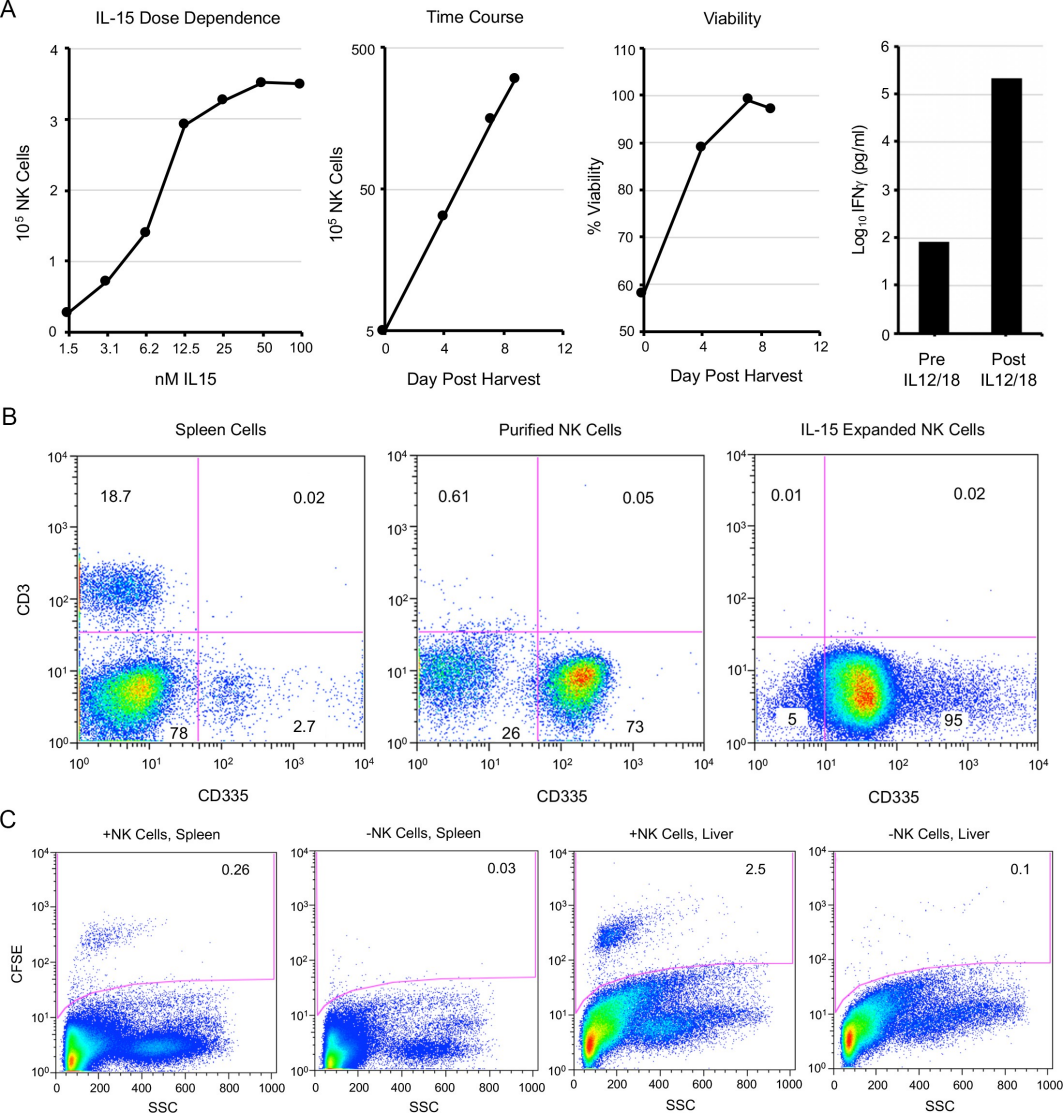
Purification and expansion of splenic NK cells. **(A)** Spleen cells from CAST mice were purified by negative selection. In left-most panel, 2-fold dilutions of IL-15 were added to purified NK cells for 5 days at 37°C and counted with a cellometer. Second panel from left shows expansion of NK cells incubated in 50 nM IL-15 over time. Third panel from left shows cell numbers and viability (trypan blue exclusion) determined with a cellometer in presence of 50 nM IL-15. Fourth panel from left shows the amounts of IFN-γ in the medium before and after incubation of expanded NK cells for 18 h with IL-12/IL-18. **(B)** Spleen cells before and after NK cell purification and following expansion with IL-15 were stained with anti-CD3 and anti-CD335 antibodies and analyzed by flow cytometry as described in the legend to Fig 1. **(C)** Purified and expanded NK cells were stained with CFSE and injected intravenously into CAST mice. After 12 h, spleen and liver cells from the mice injected with NK cells and control mice were analyzed by flow cytometry. Similar findings for CFSE were found in two independent experiments.

Because of the pleiotropic effects of IL-15 on the immune system, *in vivo* experiments to establish that NK cells are sufficient to protect CAST mice against OPXV infection is problematic. However, the demonstration that passively transferred NK cells are protective would go a long way to reaching that conclusion. Since there are too few NK cells in naive CAST mice for passive transfer, we decided to expand them *in vitro*. Spleen cells were harvested, and NK cells purified by negative NK cell selection. The cells were then incubated in medium containing IL-15. The cell numbers increased in an IL-15 dose-dependent fashion, exponentially with time, and with high viability (Fig 5A). Analysis by flow cytometry (Fig 5B) revealed that 2.7% of the spleen cells were CD3^-^CD335^+^ prior to purification. After negative NK cell selection, the percentage of CD3^-^CD335^+^ cells increased to 73%. Following 11 days of IL-15 treatment, 95% of the cells were CD3^-^CD335^+^ indicating selective amplification of NK cells (Fig 5B). CD3^+^ cells comprised 18.7% of the total spleen cells, 0.61% of the purified NK cells, and only 0.01% of the IL-15 expanded NK cells (Fig 5B). The functionality of the expanded NK cells was shown by their secretion of IFN-γ upon overnight activation with IL-12 and IL-18 (Fig 5A).

To ascertain their competence for homing to peripheral organs, the expanded NK cells were labeled with carboxyfluorescein succinimidyl ester (CFSE) and injected by the intravenous route into CAST mice. Fluorescein-labeled NK cells were detected in the spleen and liver at 12 h (Fig 5C).

### Passive transfer of NK cells is protective

**Fig 6:**
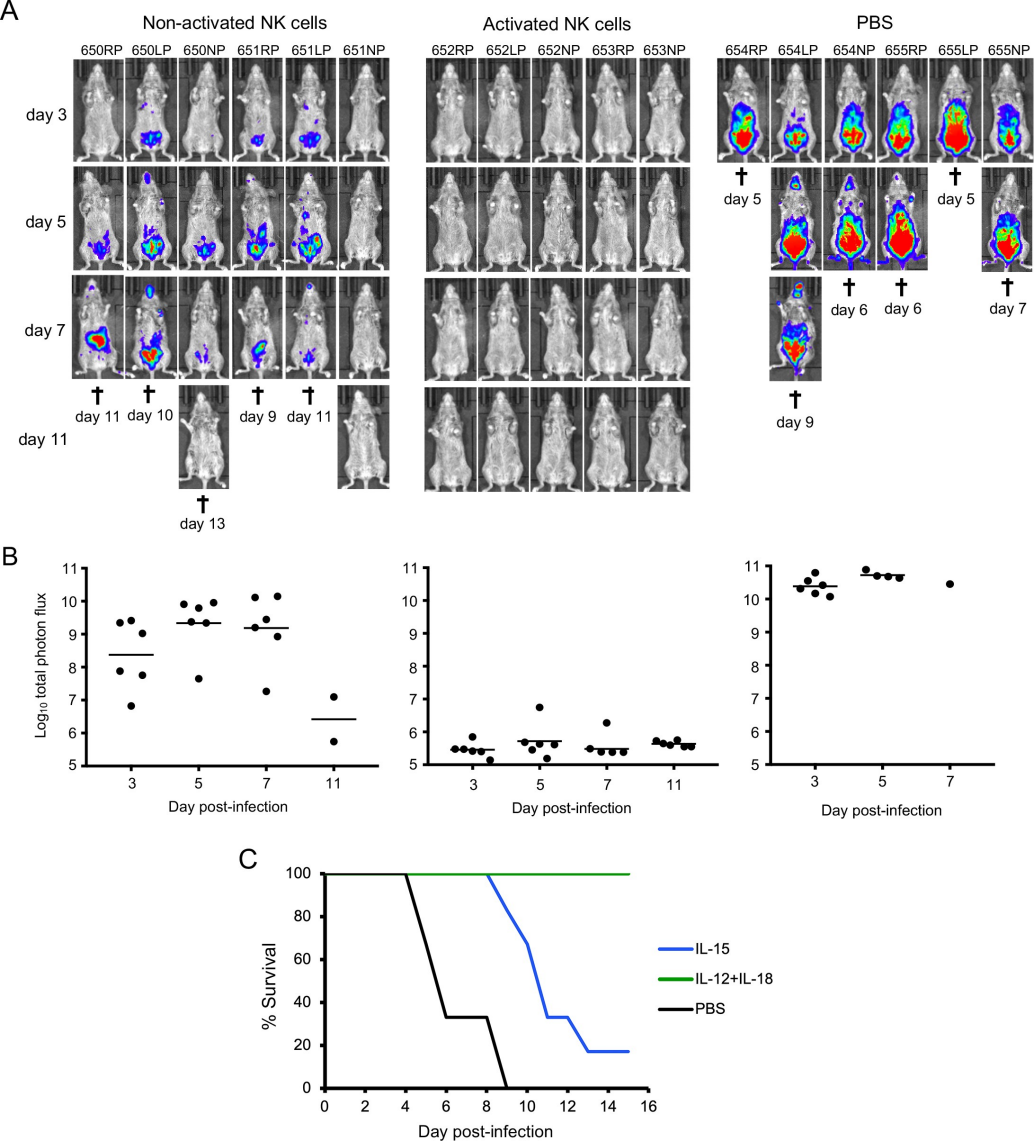
**Passive transfer of purified NK cells. (A)** CAST mice were inoculated IP with PBS or 3.5x10^6^ purified NK cells that were expanded *in vitro* with IL-15 and either non-activated or activated with IL-12/IL-18. After 4 h, the mice were challenged with 480 PFU of VACV expressing FLuc. Virus spread was determined from luminescence as described in Fig 2 and ventral images are shown. **(B)** The plots of total photon flux of individual animals from panel A are below the images for each group. **(C)** The days mice died or were euthanized (†) and survival curves are shown. Protection by NK and activated NK cells was shown in two independent experiments.

The ability to purify and expand NK cells allowed us to determine whether they are sufficient to protect CAST mice against an OPXV infection. PBS or non-activated or IL-12/IL-18 activated NK cells were washed to remove any soluble cytokines and inoculated IP into CAST mice, which were challenged 4 h later with a potentially lethal dose of VACV expressing FLuc. Animal imaging revealed rapid spread of virus (Fig 6A and 6B) leading to death (Fig 6C) in the PBS control animals between days 5 and 9. Remarkably, virus spread as determined by luminescence was minimal or undetectable in the mice that received activated NK cells and there were no deaths (Fig 6A and 6B). CAST mice that received non-activated NK cells exhibited higher virus spread than those receiving activated NK cells (p = 0.0022 for days 3–7). Nevertheless, the mice receiving non-activated NK cells had lower virus spread than the controls (p = 0.0022 and 0.0095 on days 3 and 5, respectively), though 5 of 6 died between days 9 and 13 (Fig 6A, 6B and 6C). The median time to death of mice receiving non-activated NK cells was 11 days compared to 6 days for the PBS controls (p = 0.0012, Mantel-Cox).

Because the mice that were fully protected by activated NK cells exhibited little or no evidence of virus spread, we were curious to know whether they would be resistant to re-challenge with VACV two weeks later. As controls to confirm the virulence of the virus used for the re-challenge, two naïve mice were also challenged, and both exhibited high luminescence and died on day 7 (S3A and S3B Fig). The previously protected mice exhibited low levels of luminescence upon re-challenge except for one mouse that died on day 10 (S3A and S3B Fig). Protection of the re-challenged mice could have been due to persistence of NK cells or an adaptive immune response despite evidence for little or no virus spread following the initial challenge. On the day preceding the secondary challenge, no antibodies to VACV were detected by ELISA in three of the previously protected mice and low titers in two (S3C Fig). Nevertheless, a strong adaptive immune response occurred following the second challenge as evidenced by the high ELISA titers after two weeks. A likely explanation is that despite the complete protection of mice that received activated NK cells, the initial virus challenge provided priming for a secondary adaptive immune response after a subsequent infection.

### Duration of protection following NK cell transfer

**Fig 7:**
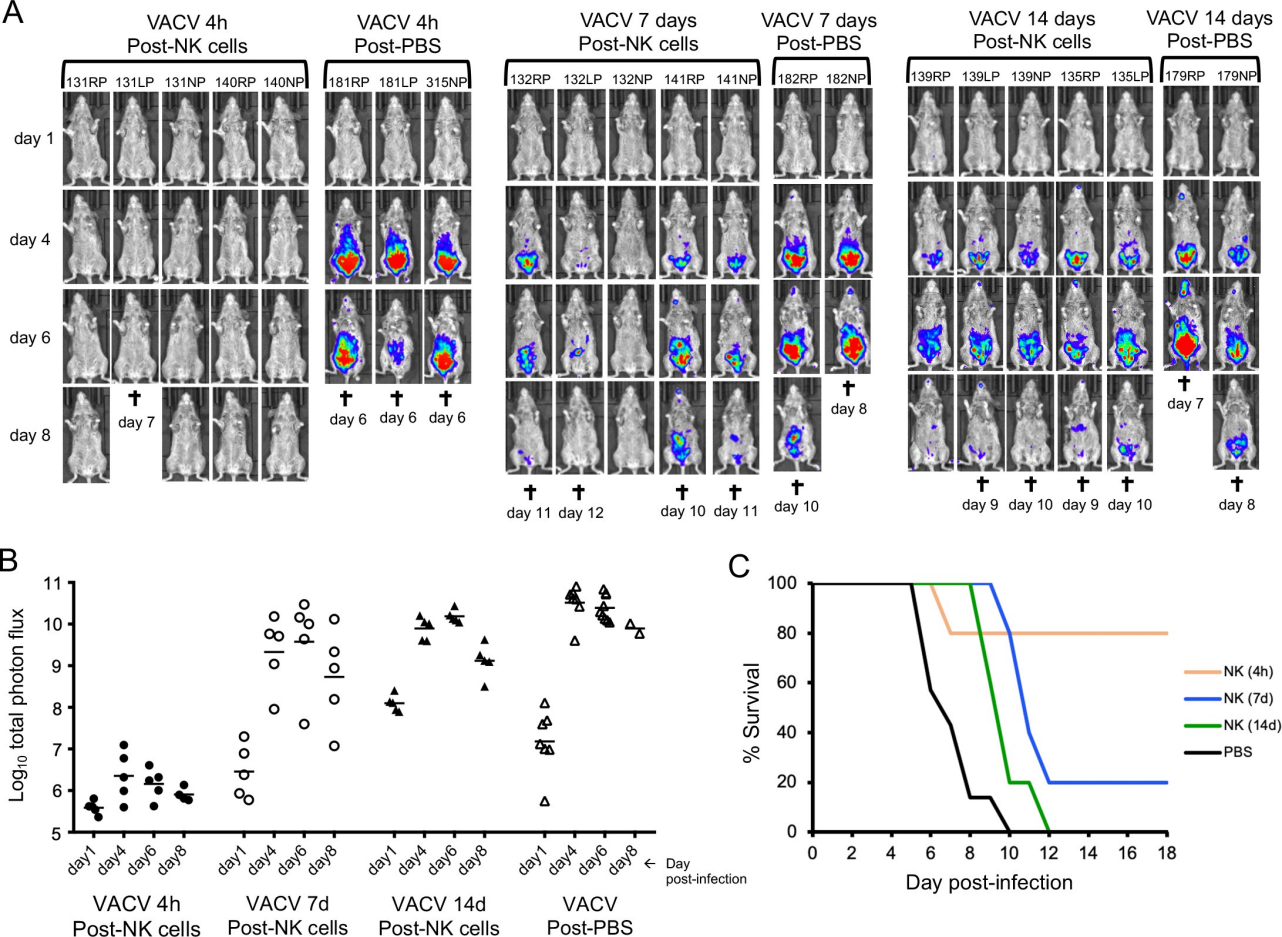
Duration of protection following passive transfer of activated NK cells. **(A)** CAST mice were inoculated with PBS or 3.1 x10^6^ activated NK cells. After 4 h, 7 days or 14 days, the mice were challenged with 550 PFU of VACV expressing FLuc. Virus spread was determined from luminescence as described in Fig 2 and ventral images are shown. **(B)** For the photon flux plot the control mice treated with PBS were grouped together. **(C)** For the survival curve the control mice treated with PBS also were grouped together.

To determine the duration of protection, activated NK cells were passively transferred to naïve mice, which were challenged after 4 h, 7 days or 14 days (Fig 7A and 7B). Mice that were challenged after 4 h exhibited low luminescence and 4 of 5 survived. Mice that were challenged after 7 or 14 days had increasingly higher luminescence than those challenged after only 4 h but still lower than the controls that received PBS (p = 0.0025, 0.0177, 0.0030 on day 4 after the 4 h, 7 day and 14 day challenges compared to PBS group, respectively). Furthermore, compared to the controls, the time to death was longer (p = 0.0021 and p = 0.021, Mantel-Cox) for the 7- and 14-day challenges, respectively (Fig 7C). These results indicated an initial rapid decline in protection that persisted at a reduced level following transfer of activated NK cells to CAST mice, consistent with a low level of NK cell homeostasis.

**Fig 8:**
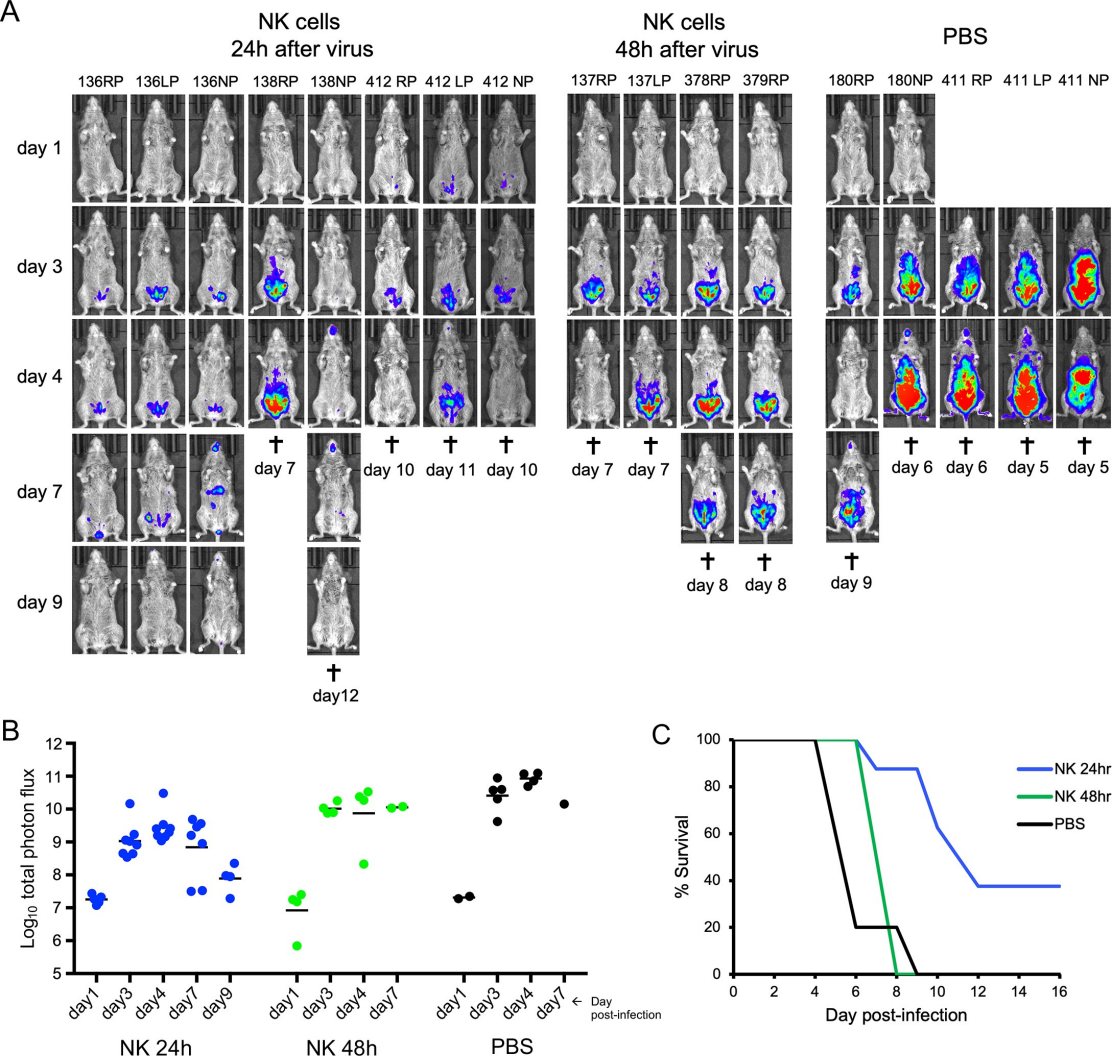
Passive transfer of activated NK cells following VACV infection. **(A)** Data are compiled from two separate experiments. In the first experiment, CAST mice were infected IP with 655 PFU of VACV expressing FLuc. After 24 or 48 h, 4 to 5 mice were inoculated IP with 3.1x10^6^ activated NK cells. Two mice received PBS instead of NK cells as a control. In the second experiment, CAST mice were infected with 550 PFU of VACV and after 24 h, 3 mice (412RP, 412LP, and 412NP) were inoculated with 3.0 X 10^6^ activated NK cells and 3 mice (411RP, 411LP and 411NP) received PBS. Virus spread was determined by luminescence as in Fig 2. Ventral images of individual mice are shown. **(B)** The total photon flux for individual animals are shown. The difference between the 8 mice receiving NK cells at 24 h after infection versus the 5 PBS controls was significant on days 3 and 4 after infection (p = 0.0031 and 0.004, respectively). (C) Days of death or euthanasia (†) and survival curves for the two experiments are shown. The time to death between the mice receiving NK cells at 24 h after infection and PBS controls was significant (p = 0.022, determined by Mantel-Cox test).

We also investigated whether passive transfer of NK cells could ameliorate a prior infection. CAST mice were challenged with lethal doses of VACV and activated NK cells were administered 24 or 48 h later. Mice not receiving NK cells that were infected as controls exhibited high luminescence (Fig 8A and 8B) and died (Fig 8C). Mice receiving NK cells at 24 h after infection had lower luminescence (Fig 8A and 8B) than the controls (p<0.004) and prolonged survival (p = 0.02) (Fig 8C). NK cells administered at 48 h after infection provided little or no protection (Fig 8C). Thus, passive transfer of NK cells following challenge can be beneficial, but the time interval for protection is short due to the rapid replication of VACV.

### Antibodies to IFN-γ diminish protection mediated by activated NK cells

**Fig 9:**
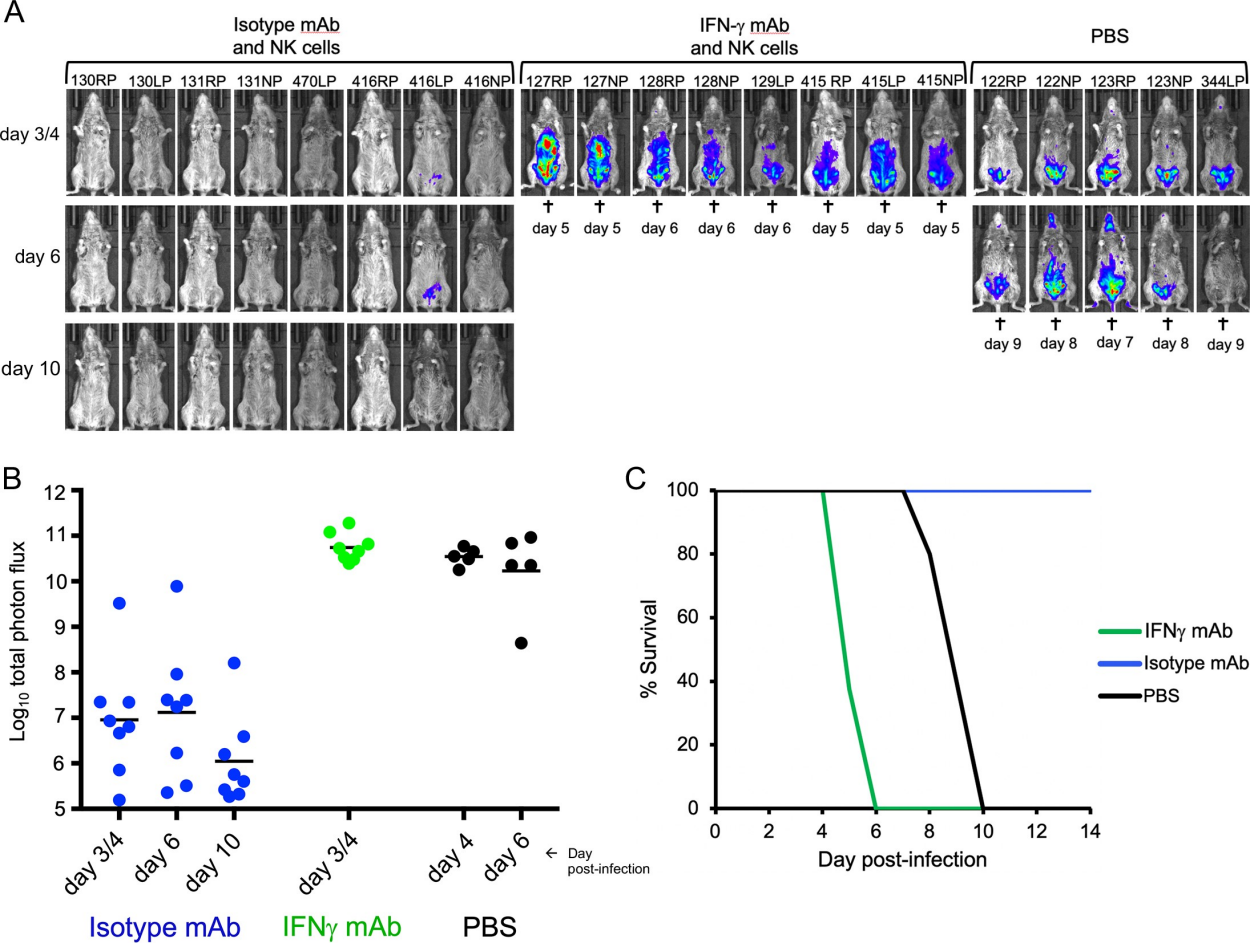
Antibodies to IFN-γ diminish protection mediated by activated NK cells. **(A)** Data are compiled from two separate experiments. CAST mice were injected IV on day -3 and IP on days -1, +1, +3, +6, +8 with a mAb specific for IFN-γ, an anti-HRP mAb (isotype control) or PBS prior to and after administration of activated and washed NK cells. After 4 h, the mice were challenged with 430 PFU of VACV expressing FLuc. Luminescence was measured as described in the legend of Fig 2. Ventral images are shown. Days of death or euthanasia (†). **(B)** Total photon flux of animals in panel A. (C) Survival curves.

NK cells suppress viral infections by multiple ways including cytolysis of infected cells and secretion of a variety of cytokines including IFN-γ, TNF-α, GM-CSF and MIP-1α/β [46, 47]. In view of our previous study demonstrating the protective effect of exogenous IFN-γ [33], we suspected that secretion of IFN-γ by the NK cells contributed to protection. To evaluate this, CAST mice were treated either with antibody specific for IFN-γ, an isotype-specific antibody, or PBS prior to and after administration of activated and washed NK cells and challenged 4 h later with a lethal dose of VACV. Mice that received NK cells and isotype Mab exhibited low levels of luminescence (Fig 9A and 9B) and all survived (Fig 9C). In contrast, those mice that received NK cells and anti-IFN-γ antibody had high levels of luminescence (Fig 9A and 9B) and died even faster than mice that received only PBS (Fig 9C). We attribute the more rapid death to the neutralization of IFN-γ synthesized by both endogenous and passively transferred NK cells.

## Discussion

Previous investigations demonstrated that naïve CAST mice have low numbers of NK cells and make a delayed IFN-γ response to infection, suggesting that an inadequate innate immune response contributes to their exceptional susceptibility to OPXVs [32, 33]. Here we provide support for this conclusion by showing that increasing NK cell numbers *in vivo* or passively transferring purified NK cells expanded *ex vivo* enhanced protection against OPXV.

We focused on IL-15, as this cytokine plays a dominant role in NK cell homeostasis and mediation of an effective anti-viral response [38, 39, 48]. Our first question was whether the NK cells of CAST mice could be increased by administration of IL-15 *in vivo*. We documented an increase in the number of NK cells in the spleen, peritoneum and liver after administering IL-15 for 4 days, informing us that CAST mice have progenitor NK cells that are capable of responding to IL-15 by rapid proliferation and peripheral localization. Moreover, a high proportion of the NK cells could express IFN-γ indicating activation by endogenous cytokines. IL-15 also increased CD8^+^ T cells but had little effect on CD4^+^ T cells and no discernible effect on NKT cells, which are present in very low numbers in CAST mice.

Having achieved an increase in NK and CD8^+^ T cell numbers, our next question was whether this treatment would protect against a potentially lethal OPXV infection of CAST mice. Virus spread was monitored by employing recombinant VACV and MPXV that express FLuc for live imaging. The IL-15-treated mice exhibited decreased spread of VACV and MPXV and increased survival following challenges between 1- and 5-days post IL-15 treatment. Although NK cells are a first line of defense that operate ahead of adaptive T cell responses, complete protection of mice against an OPXV challenge depends on both types of immune responses [44]. Therefore, further experiments were carried out to distinguish the effects of NK and T cells. We found that depletion of CD4^+^ and CD8^+^ T cells did not prevent the decrease in virus spread due to IL-15-treatment during the first week after infection but that T cells were necessary for virus clearance at later times. Although the T cell depleted mice eventually succumbed to infection, IL-15 significantly reduced virus spread and prolonged survival relative to control mice, supporting a role for the early expansion of NK cells in protection.

Passive transfer experiments were carried out to more rigorously demonstrate the role of NK cells in protection. Splenic NK cells were purified and incubated with IL-15 *in vitro* to determine whether this cytokine was sufficient for proliferation of CAST NK cells and to provide adequate numbers of highly purified NK cells for passive transfer. Analysis of the expanded population revealed 95% CD335^+^ NK cells and barely detectable CD3^+^ cells. The functionality of the NK cells was demonstrated by secretion of IFN-γ in response to IL-12 and IL-18, which together are potent inducers of IFN-γ [49–51]. The ability of the purified NK cells to home to the spleen and liver was demonstrated by labeling them with a fluorescent dye. Following passive transfer of non-activated NK cells to CAST mice, VACV replicated to lower levels than in control mice that did not receive NK cells and the median time to death was significantly increased relative to controls. NK cells activated *in vitro* with a combination of IL-12 and IL-18 provided greater protection as virus spread was not detected when the mice were challenged, and the mice all survived. The protection of CAST mice that were challenged after 4 days of IL-15 administration *in vivo* can also be attributed to activated NK cells as the majority could express IFN-γ. Activation of NK cells following infection may be limited by expression of IL-18 binding protein encoded by VACV, which has been shown to increase virulence [52]. Although NK cells can control virus infection by both IFN-γ- and perforin-mediated mechanisms, the importance of the former was demonstrated by the failure of CAST mice to be protected by activated NK cells when they received IFN-γ neutralizing antibodies. Thus, IFN-γ appears to be a common denominator of protection whether the cytokine itself or activated NK cells are administered.

Our studies indicate that the relatively low level of NK cells in naïve CAST/EiJ mice is insufficient to protect against systemic infection with OPXVs. CASA/RkJ, a strain that was independently derived from the same founder population of wild mice trapped in Thailand, is equally susceptible to OPXV [26]. Furthermore, the MOLF/EiJ strain, which was derived from mice trapped in Japan, has low numbers of NK cells and is also susceptible to MPXV [26]. Indeed, of 32 inbred mouse strains examined for the number of NK cells in the blood, CAST was number 29 and MOLF was 32 [53]. The NK cell number of PARA/EiJ, the third mouse strain susceptible to MPXV, is not available. As the wild-derived inbred mouse strains were established from a small number of founders, they may not represent the genetic diversity of the wild population or the situation when the mice are reared in nature. Despite these caveats, the low NK cell numbers of these wild-derived inbred strains are puzzling since NK cells provide an early defense against viruses. However, high numbers of NK cells can be associated with pathology and consume metabolic resources so that low numbers may be advantageous in some environments and should not necessarily be considered as defects.

The factors regulating the number of NK cells are complex and not completely understood [54]. Low numbers could arise at the level of differentiation from progenitors, which depends on cell-cell interactions between hematopoietic precursors and bone marrow stromal cells as well as soluble growth factors and cytokines [48] or as a result of diminished peripheral NK cell homeostasis, which depends on IL-15 availability and receptor interactions [38, 45, 55, 56]. Under natural conditions, stimulation of NK cells is thought to occur in trans by IL-15 bound to IL-15 receptor alpha. Low homeostatic regulation in CAST mice was suggested by our findings that the elevated numbers of NK cells following IL-15 treatment or passive transfer, declined within several days. This contrasts with data obtained with C57Bl/6 mice in which passively transferred NK cells in the spleen changed only slightly between 1.5 and 5 days [55]. The factors limiting NK cell numbers in CAST mice will be a focus of our future investigations.

## Materials and methods

### Ethics statement

All experiments and procedures using mice were approved under protocol LVD29E by the NIAID Animal Care and Use Committee according to standards set forth in the NIH guidelines, Animal Welfare Act, and US Federal Law. Euthanasia was carried out using carbon dioxide inhalation in accordance with the American Veterinary Medical Association Guidelines for Euthanasia of Animals (2013 Report of the AVMA Panel of Euthanasia).

### Cells and viruses

African green monkey BS-C-1 cells (ATCC CCL-26) obtained from the ATCC were maintained at 37°C and 5% CO_2_ in modified Eagle minimal essential medium (EMEM) (Quality Biologicals, Inc, Gaithersburg, MD) supplemented with 8% heat-inactivated fetal bovine serum (FBS), 10 U/ml penicillin, 10 mg/ml streptomycin, and 2 mM L-glutamine and used for propagation and titration of VACV and MPXV. The recombinant strain of Western Reserve VACV (WRvFire) and the Zaire-1979 strain of MPXV (MPXV Z06) expressing FLuc regulated by a synthetic early-late promoter were previously described [43, 57] and were propagated in BS-C-1 cells, purified by sedimentation through a cushion of sucrose and titrated according to previously described protocols [58].

### Mice

Female CAST/EiJ, B6:129X1-*Il15ra*^*tm1Ama*^/J and B6-129X1 (JAX: B6129SF2/J) mice were purchased from Jackson Laboratories (Bar Harbor, ME) and female C57BL/6 mice were from Taconic Biotechnology (Germantown, NY). Mice were housed in small, ventilated microisolator cages in an ABSL2 or ABSL3 pathogen-free environment.

### IL-15 stimulation of mice

Recombinant murine IL-15 was purchased from Peprotech (Rocky Hill, NJ), reconstituted in phosphate buffered saline (PBS) + 0.1% bovine serum albumin (BSA) to a concentration of 1 μg/μl and stored in small aliquots at -30C. On the day of inoculation, IL-15 was thawed, diluted to 0.05 μg/μl in PBS + 0.05% BSA and administered to mice IP in 0.1 ml. Mock-stimulated mice received 0.1 ml of PBS-0.05% BSA.

### Infection of mice

CAST mice were usually 9–13 weeks old and weighed between 10–13 g at the time of use for experiments. On the day of challenge, WRvFire or MPXV Z06 was thawed, sonicated and diluted in PBS + 0.05% BSA. Mice were either injected IP with 0.1–0.2 ml of diluted virus or mock-infected with a similar volume of PBS-0.05% BSA. A small portion of diluted virus was saved and titrated on BSC-1 cells to verify the challenge dose. The latter values are provided in the Figure Legends. Animals were observed 5–7 days per week for up to 22 days post-infection.

### Isolation of splenic, hepatic, and peritoneal lymphocytes

Spleens and livers were harvested and placed in ice-cold cRPMI consisting of RPMI-1640 medium (Quality Biologicals, Inc., Gaithersburg, MD) supplemented with 10% heat-inactivated fetal bovine serum, 10 U/ml penicillin, 10 mg/ml streptomycin, 2 mM L-glutamine, and 2 mM HEPES. Individual spleens were pulverized by Dounce homogenization to create single-cell suspensions. Erythrocytes were lysed with 2 ml of ACK Lysis Buffer (Lonza, Walkersville, MD), incubated for 2 min on ice and then washed with 3x with 50 ml portions of cRPMI. Splenocytes were passed through a 70-μm cell strainer to remove coarse debris, counted and resuspended in cRPMI. Livers were rinsed with cRPMI and then pressed through a 70-μm cell strainer with a 1-cc syringe bulb. Filtered cells were centrifuged and resuspended in 30% (v/v) Percoll in RPMI. After centrifugation, the leukocyte pellet was retained and erythrocytes were lysed with 2 ml of ACK Lysis Buffer, incubated for 5–10 min at room temperature and washed with 3x 50 ml portions of cRPMI. Lymphocytes were passed through a 70-μm cell strainer, counted and resuspended in cRPMI. To isolate peritoneal cells, peritoneal cavities of mice were washed with 2.5 ml of ice-cold cRPMI and cells were collected by centrifugation. Cell pellets were washed with 2x 50 ml portions of cRPMI. Peritoneal lymphocytes were passed through a 70-μm cell strainer to remove debris, counted and resuspended in cRPMI.

### Cell staining and flow cytometry

Single cell suspensions of lymphocytes were obtained using methods described above. Prior to cell-surface staining, Fc receptors were blocked with anti-CD16/32 (Clone 2.4G2) (gift from Jack Bennink, NIAID) for 30 min at 4°C. Cells were then stained with anti-mouse CD3e-FITC (Clone 145-2C11; Invitrogen) and anti-mouse CD335-APC (Clone 29A1.4; Biolegend) for 1 h at 4°C. Alternatively, cells were stained with anti-mouse CD3e-FITC and anti-mouse CD335-PerCP (Clone 29A1.4; BD Biosciences). NKT cells were detected with mouse CD1d PE-labeled tetramer kindly provided by the NIH Tetramer Core Facility at Emory University. Antibodies for detection of CD4^+^ (anti-mouse CD4-PE) and CD8^+^ (anti-mouse CD8a-PerCP-Cy5.5) T cells were obtained from BD Biosciences. For detection of IFN-γ production, cells were stimulated with phorbol 12-myristate 13-acetate (PMA) and ionomycin (Sigma-Aldrich, St. Louis, MO) for 15 min after which brefeldin A (Sigma-Aldrich) was added for 4–5 h. Surface staining was performed as described above and cells were then fixed and permeabilized with Cytofix/Cytoperm solution and stained with anti-mouse IFNγ-APC (both from BD Biosciences). NKT cells were detected with mouse CD1d PE-labeled tetramer provided by the NIH Tetramer Core Facility at Emory University. After staining, cells were washed 2x with PBS and resuspended in 2% paraformaldehyde. Approximately 50,000–150,000 cells were acquired on a FACS Calibur cytometer using Cell Quest software (BD Biosciences, San Jose, CA) and analyzed using Flow-Jo software (TreeStar, Cupertino, CA).

### Depletion of T-cells and IFN-γ

mAbs against CD4 (clone GK1.5), CD8a (clone YTS 169.4), or KLH (isotype control, clone LTF-2) from Bio X Cell (Lebanon, NH) were utilized for T cell depletion experiments. Mice were injected IP with 200 μg of both CD4/CD8a mAbs or 400 μg of KLH-isotype control mAb in 200 μl of PBS for four consecutive days prior to challenge with WRvFire. The efficacy of T-cell depletion was tested by flow cytometry of cells from spleens at 24 h after the last dose of depletion antibody.

mAbs directed to mouse IFN-γ (clone XMG1.2) or HRP (isotype control, clone HRPN) from Bio X cell were utilized for IFN-γ-depletion experiments. Mice were injected intravenously (IV) on day -3 and IP on days -1, +1, +3, +6 and +8 with 200 μg of IFN-γ mAb or 200 μg HRP-isotype control mAb in 200 μl of PBS on two non-consecutive days prior to challenge with WRvFire and continuing every other day up to day 5 post-infection.

### Bioluminescence imaging

Bioluminescence imaging of live animals was performed with an IVIS Lumina LT Series III system (Perkin Elmer, Waltham, MA). Infected mice were anesthetized with isoflurane and Xenolight D-luciferin substrate (Perkin Elmer, Waltham, MA) was injected intraperitoneally (150 μg/g body weight) 10 min prior to live imaging. Animals remained under isoflurane sedation for the duration of each imaging session which occurred at various timepoints post-infection until death or total viral clearance was achieved. Luminescent exposures were collected for 1–60 s with small or medium binning factors and various f-stop settings. Living Image Software (Perkin Elmer) was used for acquisition and analysis. For photon flux, a single region of interest was drawn around the entire body and light emission was measured in photons/sec/cd^2^/sr.

### Purification and expansion of NK cells

Single-cell suspensions of splenocytes were obtained using methods described above, resuspended at a concentration of 2.5x10^8^ cells/ml in autoMACS Rinsing Solution + 0.5% BSA (Miltenyi Biotech, Auburn, CA), and untouched NK cells were purified using a mouse NK cell isolation kit (Miltenyi Biotech). For expansion, isolated NK cells were grown at 37°C and 5% CO_2_ for 10–12 days in optimized NK cell medium containing cRPMI supplemented with 1X non-essential amino-acids, 50 μM β-mercaptoethanol, and 50 nM recombinant murine IL-15. Total cell number and viability were assessed using a Cellometer Auto T4 (Nexcelom, Lawrence, MA) by trypan blue exclusion and purity of the amplified culture was determined by FACS analysis after surface staining of cells with anti-mouse CD3e-FITC and anti-mouse CD335-PerCP at various times post-harvest.

### NK cell activation and passive transfer of NK cells

For NK cell activation, IL-12p70 and IL-18 were purchased from Peprotech (Rocky Hill, NJ) and MBL International (Woburn, MA), respectively. IL-15-amplified NK cells were stimulated for 18 h with 1.3 nM IL-12 and 22.2 nM IL-18 in optimized NK cell medium. As a non-activated control, a portion of NK cells were incubated with 50 nM IL-15 in optimized NK cell medium. After overnight stimulation, a small sample of supernatant was removed from both IL-12/18 and IL-15 cultures and IFN-γ levels were quantitated by xMAP cytokine bead array (EMD Millipore, Billerica, MA) using a BioPlex 200 Array Reader (Bio-Rad, Hercules, CA). NK cells were washed with 2x 50 ml portions of PBS and resuspended to a final concentration of 1–1.5x10^7^ cells/ml in PBS + 0.05% BSA. Groups of CAST/EiJ were inoculated IP with 3–3.5x10^6^ cells in 0.2 ml.

For NK cell-labeling experiments, IL-12/IL-18 activated NK cells were washed 2x with 50 ml of PBS and resuspended to a final concentration of 5x10^6^ cells/ml in 1 μM CFSE (Thermo Fisher Scientific, Waltham, MA) in PBS. After staining for 10 min in the dark at room temperature, CFSE labeling was quenched by addition of ice-cold cRPMI. Cells were washed with 50 ml of PBS and resuspended to a final concentration of 1–1.5x10^7^ cells/ml in PBS + 0.05% BSA. Groups of CAST/EiJ were inoculated IV with 2-3x10^6^ cells in 0.2 ml.

### VACV ELISA

Total IgG titers from sera of naïve and VACV-infected mice were determined as previously described [28].

## Supporting information

S1 Fig Enumeration of NKT (CD3^+^CD1d^+^) cells in CAST mice. Mice were inoculated IP with PBS or PBS containing 5 μg of IL-15 daily for 4 days and splenic cells were isolated and stimulated with PMA/Ionomycin for 4 h, treated with Brefeldin A and stained with antibody for cell surface markers (anti CD3-FITC and anti-CD1d-PE). **(A)** Total CD3^+^ cells, **(B)** percent of lymphocytes that are CD3^+^, **(C)** total CD3^+^CD1d^+^ cells, **(D)** percent of CD3^+^ cells that are CD3^+^CD1d^+^.
(TIF)

S2 Fig MPXV challenge of IL-15-treated CAST mice. PBS alone or with 5 μg of IL-15 was administered IP to CAST mice on four successive days. The mice were infected IP with 92 PFU of MPXV on the day following cessation of treatment. The progress of infection was measured by injecting luciferin and measuring luminescence. **(A)** Ventral view images of surviving mice are shown on indicated day following infection using the same exposure times and bin. Purple, blue and red denote intensity of luminescence from low to high. Days of death or euthanasia are indicated by †. Death of one mouse occurred on day 7 after imaging. **(B)** Total photon flux (photons per second per square centimeter per steradian) of entire animals were calculated and shown for each individual mouse.
(TIF)

S3 Fig Re-challenge of NK cell-protected mice. **(A)** The 5 CAST mice that received activated NK cells shown in Fig 6 were re-challenged after 14 days with 590 PFU of VACV expressing FLuc. Two new naïve mice were also challenged to serve as controls for virus infectivity. Note that both naïve mice died on day 7, one before and one after imaging. Luminescence was measured as described in the legend of Fig 2. **(B)** Total photon flux for NK cell protected and naïve animals was calculated on days 3, 5, 7 post-infection. (C) VACV ELISA titers for total IgG were determined on sera from naïve uninfected mice and on the NK cell protected mice from panel A on day 13 prior to re-challenge and after an additional 13 days.
(TIF)
