# Supplementary figures and images for: Natural killer cells expanded in vivo or ex vivo with IL-15 overcomes the inherent susceptibility of CAST mice to lethal infection with orthopoxviruses
Source: PLoS Pathog. 2020 Apr 22;16(4):e1008505. doi: 10.1371/journal.ppat.1008505 (PMC7197867; doi:10.1371/journal.ppat.1008505)

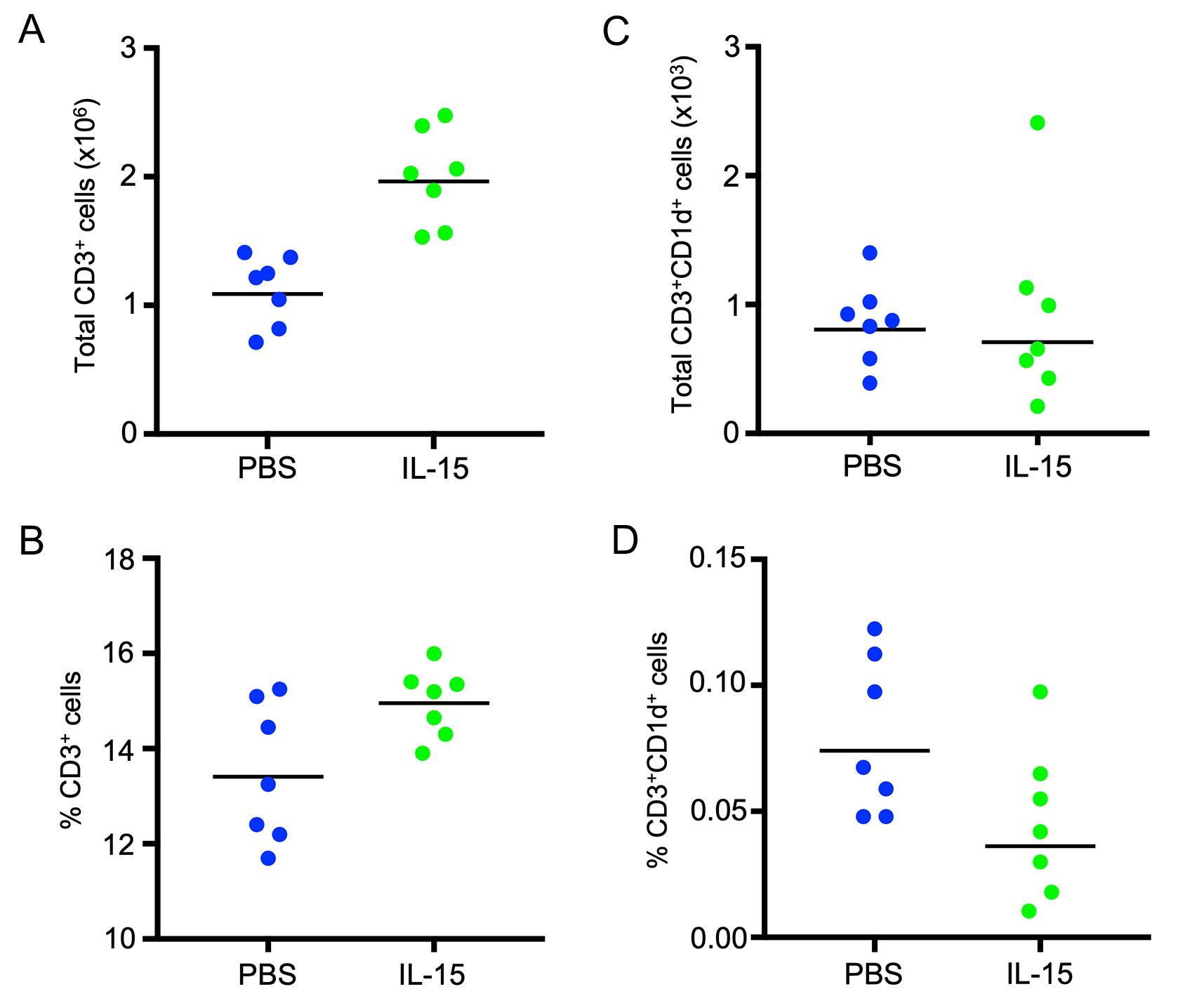

Supplement: S1 Fig — Mice were inoculated IP with PBS or PBS containing 5 μg of IL-15 daily for 4 days and splenic cells were isolated and stimulated with PMA/Ionomycin for 4 h, treated with Brefeldin A and stained with antibody for cell surface markers (anti CD3-FITC and anti-CD1d-PE). (A) Total CD3+ cells, (B) percent of lymphocytes that are CD3+, (C) total CD3+CD1d+ cells, (D) percent of CD3+ cells that are CD3+CD1d+. (TIF) [file ppat.1008505.s001.tif]

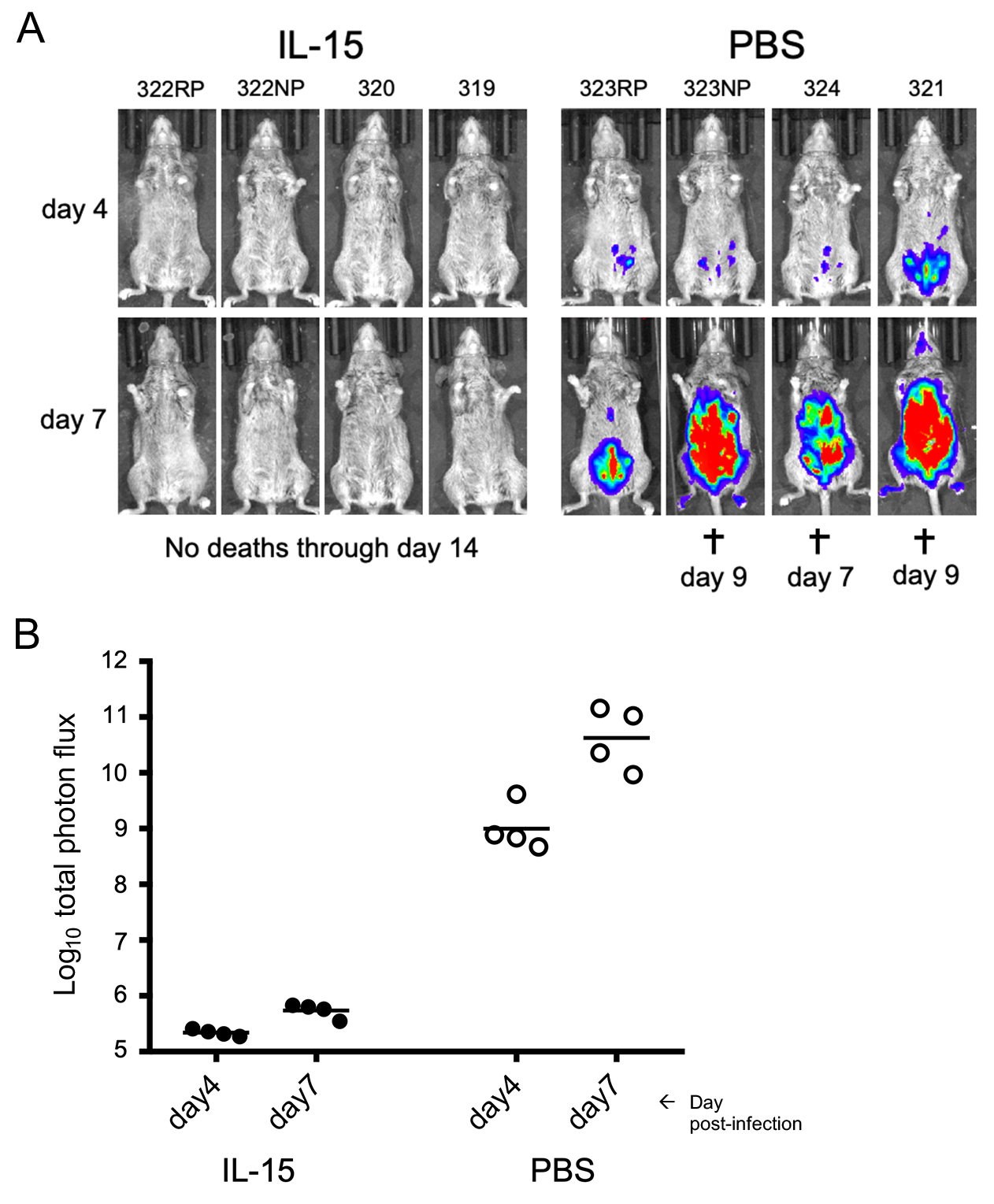

Supplement: S2 Fig — PBS alone or with 5 μg of IL-15 was administered IP to CAST mice on four successive days. The mice were infected IP with 92 PFU of MPXV on the day following cessation of treatment. The progress of infection was measured by injecting luciferin and measuring luminescence. (A) Ventral view images of surviving mice are shown on indicated day following infection using the same exposure times and bin. Purple, blue and red denote intensity of luminescence from low to high. Days of death or euthanasia are indicated by †. Death of one mouse occurred on day 7 after imaging. (B) Total photon flux (photons per second per square centimeter per steradian) of entire animals were calculated and shown for each individual mouse. (TIF) [file ppat.1008505.s002.tif]

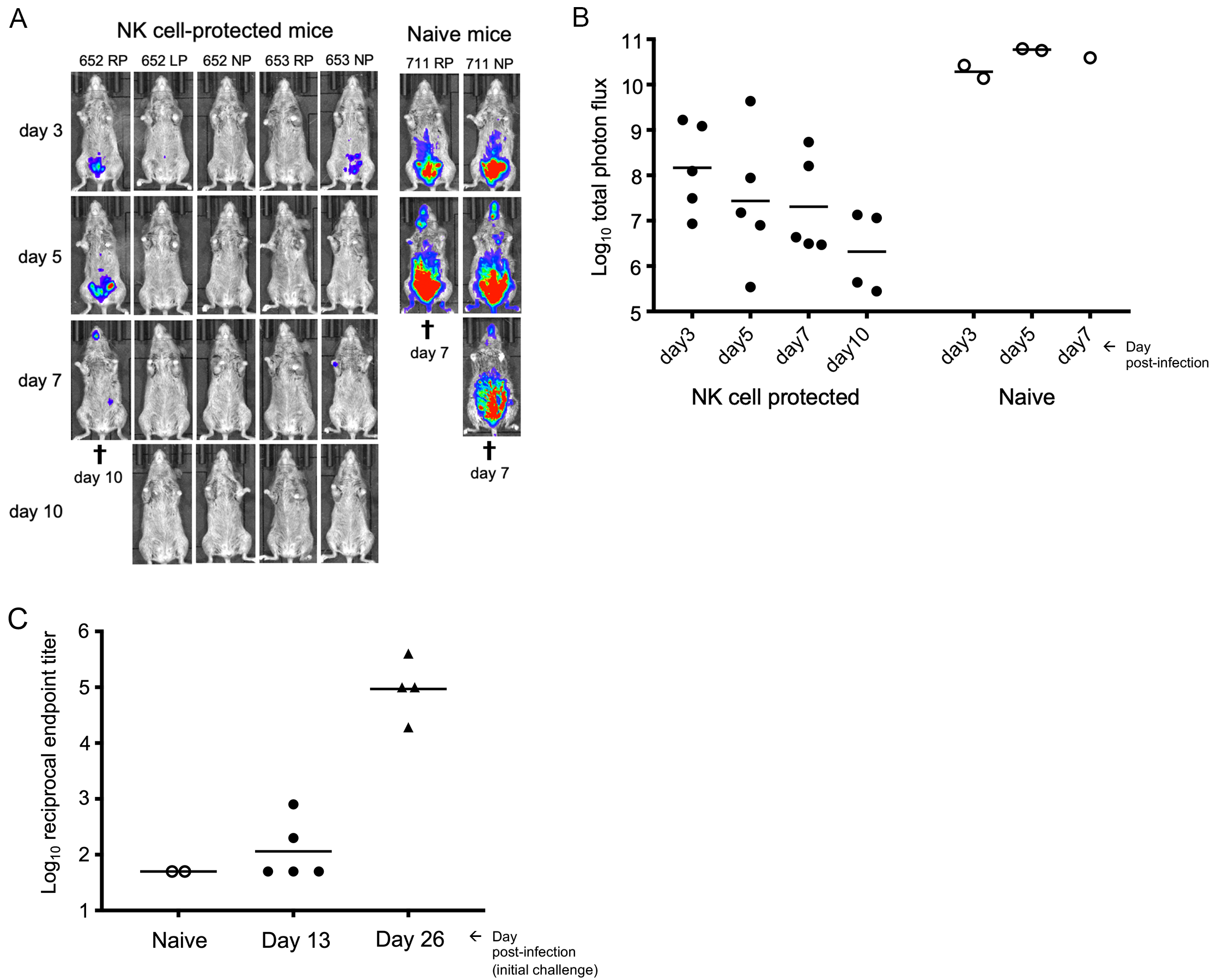

Supplement: S3 Fig — (A) The 5 CAST mice that received activated NK cells shown in Fig 6 were re-challenged after 14 days with 590 PFU of VACV expressing FLuc. Two new naïve mice were also challenged to serve as controls for virus infectivity. Note that both naïve mice died on day 7, one before and one after imaging. Luminescence was measured as described in the legend of Fig 2. (B) Total photon flux for NK cell protected and naïve animals was calculated on days 3, 5, 7 post-infection. (C) VACV ELISA titers for total IgG were determined on sera from naïve uninfected mice and on the NK cell protected mice from panel A on day 13 prior to re-challenge and after an additional 13 days. (TIF) [file ppat.1008505.s003.tif]
